# Language delay and poorer school performance in children of mothers with inadequate iodine intake in pregnancy: results from follow-up at 8 years in the Norwegian Mother and Child Cohort Study

**DOI:** 10.1007/s00394-018-1850-7

**Published:** 2018-11-12

**Authors:** Marianne H. Abel, Ragnhild E. Brandlistuen, Ida H. Caspersen, Heidi Aase, Liv E. Torheim, Helle Margrete Meltzer, Anne Lise Brantsaeter

**Affiliations:** 1 Division of Infection Control and Environmental Health, Department of Exposure and Environmental Epidemiology, Norwegian Institute of Public Health, Skøyen, P.O. Box 222, 0213 Oslo, Norway; 2 Department of Nursing and Health Promotion, Faculty of Health Sciences, Oslo Metropolitan University, St. Olavs plass, P.O. Box 4, 0130 Oslo, Norway; 3 Department of Nutrition, Tine, SA, P.O. Box 25, 0051 Oslo, Norway; 4 Division of Mental and Physical Health, Norwegian Institute of Public Health, Skøyen, P.O. Box 222, 0213 Oslo, Norway

**Keywords:** Iodine, Pregnancy, Dietary supplements, Neurodevelopment, Norwegian Mother and Child Cohort Study, MoBa

## Abstract

**Purpose:**

Some studies indicate that mild-to-moderate iodine deficiency in pregnant women might negatively affect offspring neurocognitive development, including previous results from the Norwegian Mother and Child Cohort study (MoBa) exploring maternally reported child development at age 3 years. The aim of this follow-up study was to investigate whether maternal iodine intake in pregnancy is associated with language and learning at 8 years of age.

**Methods:**

The study sample includes 39,471 mother–child pairs participating in MoBa with available information from a validated food frequency questionnaire covering the first half of pregnancy and a questionnaire on child neurocognitive development at 8 years. Multivariable regression was used to explore associations of iodine intake from food and supplements with maternally reported child outcomes.

**Results:**

Maternal iodine intake from food less than ~ 150 µg/day was associated with poorer child language skills (*p*-overall = 0.013), reading skills (*p*-overall = 0.019), and writing skills (*p*-overall = 0.004) as well as poorer school test result in reading (*p* < 0.001), and increased likelihood of the child receiving special educational services (*p*-overall = 0.042) (in non-iodine supplement users). Although significant, differences were generally small. Maternal use of iodine supplements in pregnancy was not significantly associated with any of the outcomes.

**Conclusions:**

Low habitual iodine intake in pregnant women, i.e., lower than the recommended intake for non-pregnant women, was associated with mothers reporting poorer child language, school performance, and increased likelihood of special educational services. We found no indications of benefits or harm of using iodine-containing supplements in pregnancy. Initiating use in pregnancy might be too late.

**Electronic supplementary material:**

The online version of this article (10.1007/s00394-018-1850-7) contains supplementary material, which is available to authorized users.

## Introduction

Iodine is an essential micronutrient incorporated into the thyroid hormones thyroxine (T4) and triiodothyronine (T3). Thyroid hormones are vital in the regulation of early brain development, and the developing foetus is vulnerable to low maternal T4, especially before the foetal thyroid starts to function in gestational week (GW) 18–20 [1]. Iodine status is an important determinant of risk for thyroid disorders and, as a result, abnormal thyroid hormone concentrations [2–4]. In mild-to-moderate iodine deficiency (ID), a range of autoregulatory mechanisms are triggered in the maternal thyroid to maintain the production and release of the hormones [5]. To conserve iodine, more T3 is released and less T4. The resulting maternal hypothyroxinaemia (i.e., low/suboptimal T4) may reduce foetal supply of the hormones since the direct transfer of maternal T3 over the placenta is extremely low [1].

Globally, ID is one of the most prevalent nutrient deficiencies, common in both low and high income countries [6]. Substantial efforts have been made to eradicate ID, and today the number of countries with severe ID populations has declined [7]. Still, mild-to-moderate ID is prevalent in many regions, and particularly in pregnant women, as the recommended intake in pregnancy is higher. The iodine requirement increases in pregnancy due to a higher production of thyroid hormones, an increase in renal iodide clearance, and trans-placental iodine transfer [8, 9]. In 2007, the World Health Organisation (WHO) reported that an estimated 50% of the population in continental Europe remains mildly iodine deficient [10].

Results from animal studies and observational human studies, including the Norwegian Mother and Child Cohort Study (MoBa), indicate that maternal mild-to-moderate ID might affect thyroid function in pregnancy [11], and negatively affect foetal neurodevelopment including reduced IQ, school performance, language delay, and behaviour problems [1, 12–17]. When children do not reach their full developmental potential due to ID, this is not only important for the children’s future, but also costly at a societal level [18].

Although studies indicate negative effects, there is little knowledge about what is the optimal iodine intake to secure an adequate iodine status in pregnant women. Thus, different recommendations for daily intake during pregnancy exist. In the Nordic countries, 175 µg/day is recommended [19], whereas the European Food Safety Authority recommendation is 200 µg/day [20], and the WHO recommendation is 250 µg/day [21]. Additionally, studies show conflicting results as to whether iodine supplement use initiated in pregnancy is beneficial or potentially harmful in mild-to-moderate ID populations [22]. Iodine supplements can reduce the risk of thyroid enlargement due to ID, but some studies, including results from MoBa, indicate that a sudden increase in iodine intake may temporary inhibit thyroid hormone production and cause lower availability of T4 to the foetus [11, 23, 24]. In MoBa, we observed an increased risk of child ADHD symptoms and diagnosis at age 6–13 years in children of mothers who had initiated use of iodine-containing supplements in the first trimester [13], but no effect on language and motor skills at age 3 years [12].

In this follow-up of the MoBa-children, the main aim was to investigate associations between maternal iodine intake in pregnancy (from food or from supplements) and child language and learning at age 8 years. A second aim was to evaluate current iodine status in a subsample of 300 8-years-old children.

## Materials and methods

### Subjects and design

**Fig. 1:**
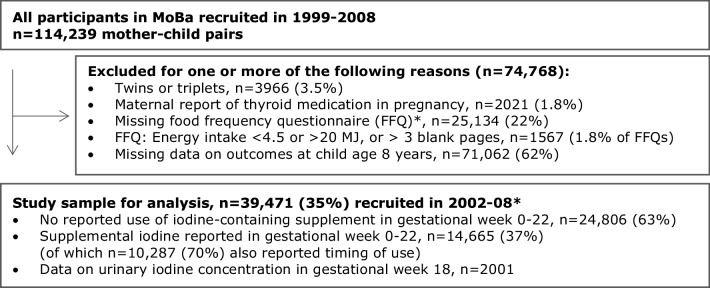
Flow chart of inclusion. *The MoBa food frequency questionnaire was introduced in March 2002

This study is based on the Norwegian Mother and Child Cohort Study (MoBa) conducted by the Norwegian Institute of Public Health [25]. Women pregnant in their first trimester were recruited from all over Norway during the years 1999–2008 and were asked to answer questionnaires (in Norwegian) at regular intervals during pregnancy and after birth. More than 99% of participants are of Caucasian origin. Pregnancy and birth records from the Medical Birth Registry of Norway are linked to the MoBa database [26]. The women consented to participation in 41% of the pregnancies. The cohort now includes 114,500 children, 95,200 mothers and 75,200 fathers. The current study is based on version 10 of the quality-assured data files released for research in 2017 and includes participants with data from the 8-year questionnaire (*n* = 39,471). A flow chart of inclusion is illustrated in Fig. 1. A subsample of 8-years-old MoBa-children living in Oslo and the surrounding areas were recruited for an EU-project [27] and contributed with urine samples for analysis of UIC (*n* = 279).

### Exposure variable: iodine intake

The MoBa FFQ [28] was specifically designed for the MoBa study and in use from 2002. It is a semi-quantitative questionnaire designed to capture dietary habits and use of dietary supplements during the first half of pregnancy [29] and was completed by participating women around GW 22. It included questions about intake of 255 food items or dishes. The average intake of specific foods and nutrients in GW 0–22 were calculated based on standard Norwegian portion sizes, the Norwegian food composition table, analyses of Norwegian food samples [30, 31], and data on the content of more than 1000 food supplements collected from suppliers [32]. There is limited data on dosage of supplements since the wording of the question on supplement use in the MoBa FFQ was unclear as to which time period it referred to (i.e., average intake in GW 0–22, intake only when using, or current use in GW 22). Therefore, we decided not to use the dosage of supplement as an exposure variable, but instead included it as a dichotomous variable (use/no use in GW 0–22).

As reported previously [12], the MoBa FFQ has been shown to be a valid tool for ranking pregnant women according to high and low intakes of energy, nutrients and foods [33]. Iodine was validated separately and iodine intake by the FFQ, including supplemental iodine, agreed well with the reference methods 24-h UIE and 4 days weighed-food diary, triangular validity coefficient for total iodine intake by the FFQ was 0.62 (95% CI 0.46, 0.77) [34, 35].

Timing of iodine supplement use was reported in the general questionnaires answered in GW 17 and GW 30. Timing of the first reported use was coded in four categories (never, week 0–26 before pregnancy, GW 0–12 and GW 12–22).

Blood and spot-urine samples were collected at the routine ultrasound examination offered free of charge to all Norwegian women in GW 18, and urinary iodine concentration (UIC) was measured for a subsample of the women (*n* = 2001) as an alternative measure of iodine intake.

UIC was also measured in a subgroup of 8-year-old children in MoBa residing in the Oslo area (*n* = 279, year of sampling 2014–2015) in a urine sample where equal volumes of an evening urine and the following morning urine were mixed.

### Outcome variables: child language and learning

Outcomes were based on the MoBa questionnaire completed by the mothers at child age 8 years. The questionnaire was distributed to all participants in MoBa by mail and the response rate was 38%.

#### Maternally reported language skills

A standardized score was calculated based on items from The Children’s communication checklist-Short (CCC-S), a brief version of the CCC-2 [36]. The full CCC-2 is as effective as a standardized assessment in identifying children with clinically significant language impairment [37]. The CCC-S contains 13 items that best discriminated typically developing children from peers with language impairment in the validation study [38], with high degrees of internal consistency (Cronbach’s *α* = 0.80, this sample) and a significant correlation between CCC-S and CCC-2 total scores in the standardization sample, Pearson’s *r* = 0.88. Each item provides an example of language behaviour in everyday contexts and covers speech, vocabulary, grammar and discourse.

#### Maternally reported child reading and writing skills

Standardized scores were calculated based on three items on reading skills: (1) reads simple stories aloud, with ease, when asked, (2) identifies all lowercase and uppercase printed letters of the alphabet, (3) reads and understands texts suitable for 7–8 years olds, and two questions on writing skills: (1) writes simple information/messages at least three sentences long, (2) writes reports, papers, or essays at least one page long; may use computer. May make small errors in spelling or sentence structure. Items were selected from the written sub-scale of the Vineland Adaptive Behaviour Scale-II [39]. Mothers answered “yes”, “partially”, or “not yet” which was coded as 0, 1, and 2.

#### Mandatory mapping tests in reading and mathematics in first and/or second grade in school

Performance tests in reading, writing, and mathematics are conducted in first and second grade. The tests map the ability to write letters, recognize written letters, identify spoken letters, combine sounds, write words, read words and read sentences. The mathematics test maps the ability to count, to compare numbers, to rank numbers, to recognize sequences of numbers, to count forward and backward from a given number, to split a number into two other numbers, to solve textual assignments and to add two numbers. Mothers were asked if they were informed by the teacher about their child’s performance on the mapping tests. Alternative answers were “masters subject well”, “must work more but teacher is not concerned”, “teacher is concerned”, or “don’t know/not discussed with teacher”.

#### Special education

Child granted special education in school (extra educational services) due to disabilities or learning difficulties was reported by the mother (yes/no).

### Covariates

Covariates were included in the models based on previous knowledge and directed acyclic graphs (DAGs). Data on covariates were obtained from different sources: maternal age and child sex was obtained from the Medical Birth Registry of Norway. Maternally reported pre-pregnancy body weight and height for the calculation of body mass index (BMI), maternal education (≤ 12, 13–16, ≥ 17 years), parity (previous pregnancies ≥ 22 weeks: 0, 1, ≥ 2), maternal chronic illness [asthma, diabetes, inflammatory bowel disease, rheumatic disease, epilepsy, multiple sclerosis or cancer before or during pregnancy (yes/no)], bilingual parent(s) (yes/no), and use of a folic acid supplement within the interval from 4 weeks before to 8 weeks after conception (yes/no) were included from questionnaire 1. Energy intake, fibre intake (as a marker of a healthy dietary pattern), and intake of the omega-3 fatty acids EPA and DHA were calculated based on the FFQ. Information on smoking in pregnancy was obtained from questionnaire 1 and, if available, questionnaires 3 (GW 30) and 4 (child’s age 6 months) (three categories: no reported smoking in pregnancy, reported occasional smoking or stopped smoking before GW 12, and daily smoking at any time in pregnancy and had not stopped smoking before GW 12). Maternal history of reading or writing difficulties (yes/no) was retrieved from the 8 years questionnaire. Paternal history of reading and writing difficulties is not reported in MoBa. Child ADHD symptoms was defined as the mean score on 18 items from the ADHD Rating Scale included in the 8-year questionnaire.

### Laboratory procedures

UIC was determined at the National Institute for Health and Welfare in Helsinki, Finland by inductively coupled plasma–mass spectrometry using an Agilent 7800 ICP-MS system (Agilent Technologies Inc, Santa Clara, CA, USA). The limit of quantification was 2 µg/L and the linearity was excellent up to 1500 µg/L (*r* = 0.99). Coefficient variation was 2–3%.

### Statistics

Statistical analyses were performed in STATA (version 15.0; Stata Corp., College Station, TX).

In total, 4.2% of the women had missing values on pre-pregnancy BMI (*n* = 956, 2.4%), maternal education (*n* = 839, 2.1%), or on all covariates from questionnaire 1 (*n* = 107, 0.3%) including parity, marital status, chronic illness, bilingual parent(s), and folic acid supplement use. Missing values for covariates were imputed using multiple imputation by chained equations in STATA, and 20 imputed datasets were generated for analyses.

Associations were estimated by generalized linear regression (gamma family) for the continuous outcomes (language-, reading-, and writing scores), logistic regression for the dichotomous outcome special education, and ordered logistic regression for the ordinal outcomes (mandatory school mapping tests in reading and mathematics). Associations between iodine from food and the outcomes were modelled flexibly by restricted cubic splines (four knot positions, at percentiles 5, 35, 65, and 95). All models were adjusted for random effects of sibling clusters since some women participated with more than one pregnancy.

Adjusted models included the following covariates based on a causal diagram (Supplementary Figure S1, Online Resource 1): maternal age, education, parity, pre-pregnancy BMI, energy intake, fibre intake, and smoking in pregnancy. Models with continuous outcomes also included the following covariate(s) to increase the precision of the estimates: child sex, bilingual parent(s) (for the language outcome), and maternal history of reading/writing difficulty (for read/write scores). To isolate the effect of long-term/habitual iodine intake, we restricted analysis on associations between iodine intake from food and outcomes to participants who had not reported any use of supplemental iodine in gestational week 0–22.

The potential impact of iodine supplement use was explored by including an interaction-term between iodine from food (modelled by restricted cubic splines) and (1) any supplement use in GW 0–22 and (2) timing of first reported use (never, initiation 1–26 weeks before conception, gestational week 0–12, or gestational week 13–22). If interactions were not significant, iodine from food was not included in the final models. Adjusted models on supplement use additionally included the covariates total intake of the n-3 fatty acids EPA and DHA (from food and dietary supplements), and maternal use of folic acid supplements within the interval from 4 weeks before to 8 weeks after conception.

Associations between maternal UIC (in µg/L) and child outcomes were also explored in multivariable regression models.

*p* values are reported for overall associations between continuous exposures and outcomes (testing H0: no association) by testing the coefficients of all spline transformations equal to zero. The tests for non-linearity were performed by testing the coefficients of the second and third spline transformations equal to zero. Potential interactions were explored by testing all interaction coefficients equal to zero. All statistical tests were performed on the imputed datasets (*n* = 20).

Results are reported including robust 95% confidence intervals (CI). A *p* value < 0.05 was considered statistically significant. Sensitivity analysis included adjusting for score on 18 items from the ADHD symptoms checklist to see if the associations were also independent of effect on ADHD symptom score at age 8 years reported in a previous publication [13].

## Results

Characteristics of the study population are shown in Table 1, and calculated iodine intake and UIC by background characteristics are shown in Supplementary Table S1 (Online Resource 1). At a group level, maternal iodine intake in pregnancy was insufficient according to WHO guidelines (i.e., iodine intake was < 250 µg/day and median UIC < 150 µg/L) [21], and also according to EFSA and Nordic guidelines [19, 20]. The calculated median iodine intake from food was 122 µg/day (IQR 89, 161 µg/day), and median UIC (mean GW 18.5 (SD 1.3)) was 67 µg/L. Women who reported use of iodine-containing supplements at the time of UIC sampling had higher UIC (median 95 µg/L vs. 59 µg/L in non-users, *p* < 0.001). Median iodine intake from food differed only slightly by background characteristics, and did not differ between supplement users (37%) and non-supplement users (63%), between women with and without UIC measurements, or between responders and non-responders to the questionnaire at 8 years (Table 1). The major dietary sources of iodine were milk/yoghurt (47%), other dairy products (13%), and lean fish (14%). Drinking water only contributed with 2%, egg with 4%, fatty fish with 4%, and all other foods with the remaining 17%. Food sources of iodine by categories of iodine intake from food are shown in Supplementary Figure S2 (Online Resource 1). Iodine supplement use was more commonly reported in nulliparous (43%), higher educated (40%), and high-income households (40%) than in primi- or multiparous, less highly educated and lower-income groups (all *p* < 0.001), but the differences were generally small. UIC was higher in supplement users (*p* < 0.001), but did not differ significantly by any other background characteristic (Supplementary Table S1, Online Resource 1). The Spearman correlation between iodine intake by the FFQ and UIC (in µg/g creatinine) was *r* = 0.31 (95% CI 0.26, 0.36).

**Table 1:** Descriptive characteristics of the study population (*n* = 39,471 mother–child pairs) by maternal iodine intake

	Iodine from food, non-supplement users			Iodine supplement use in pregnancy (week 0–22)		All	Whole MoBa^a^
	< 100 µg/day	100–150 µg/day	> 150 µg/day	No	Yes		
Study sample, *n* (%)	8096 (21)	9126 (23)	7584 (19)	24,806 (63)	14,665 (37)	39,471 (100)	101,784
Maternal age at delivery, mean (SD), years	30.5 (4.4)	30.8 (4.3)	30.6 (4.5)	30.6 (4.4)	30.6 (4.4)	30.6 (4.4)	30.2 (4.6)
Pre-pregnancy BMI, mean (SD), kg/m^2^	24.1 (4.2)	23.8 (4.0)	23.9 (4.0)	23.9 (4.0)	23.7 (4.1)	23.8 (4.1)	24.0 (4.3)
Parity, %							
0	46	42	41	43	54	47	47
1	37	37	37	37	32	35	36
2 or more	17	20	22	19	13	17	18
Maternal education, %							
≤ 12 years	27	24	27	26	22	24	32
13–16 years	43	45	46	45	45	45	41
> 16 years	28	29	25	27	31	29	25
Other/missing	2.5	1.9	2.1	2.2	2.1	2.1	2.1
Married/cohabitant, %	97.1	97.5	96.9	97.2	96.9	97.1	96.1
Smoking in pregnancy, %							
Occasionally	15	14	14	14	15	14	17
Daily	4.1	3.4	4.0	3.8	3.0	3.5	5.7
Chronic illness, %	11.1	8.7	8.4	9.4	10.8	9.9	10.2
Household income, %							
Low	24	24	27	25	23	24	29
Medium	41	42	43	42	41	42	41
High	33	32	27	31	34	32	28
Missing	2.3	2.1	2.9	2.4	2.1	2.3	3.1
Bilingual parent(s), %	9.8	9.5	7.6	9.0	10.8	9.7	10.8
Maternal history of reading/writing difficulties (%)	6.2	4.8	5.4	5.4	5.4	5.6	–
Child sex boy (%)	51.4	51.1	51.5	51.3	50.2	50.9	51.2
Iodine from food, median (IQR), µg/day	78 (63, 89)	123 (111, 136)	182 (163, 212)	122 (90, 161)	121 (89, 160)	122 (89, 161)	121 (89, 161)^b^
UIC, median (IQR), µg/L^c^	47 (26, 85)	62 (33, 105)	70 (39, 116)	59 (32, 101)	83 (43, 138)	67 (35, 115)	–
Urinary creatinine, median (90% range) g/L^c^	0.75 (0.17, 2.00)	0.76 (0.16, 1.88)	0.72 (0.21, 1.89)	0.75 (0.17, 1.92)	0.72 (0.16, 1.87)	0.74 (0.17, 1.90)	–
UIC, median (IQR), µg/g creatinine^c^	69 (44, 97)	83 (58, 115)	99 (72, 132)	83 (56, 115)	116 (75, 187)	91 (63, 139)	–
Iodine supplement (%)	0	0	0	0	100	37	37^b^
Folic acid supplement (%)^d^	71	71	68	70	84	75	69
Omega 3 supplement (%)	79	81	82	81	80	80	79^b^
Maternal energy intake, median (IQR), MJ	8.0 (6.9, 9.3)	9.2 (8.1, 10.6)	11.0 (9.6, 12.8)	9.3 (7.9, 11.0)	9.3 (7.9, 11.0)	9.3 (7.9, 11.0)	9.4 (7.9, 11.1)

^a^Includes all pregnancies in the Norwegian Mother and Child Cohort Study with information on background characteristics from questionnaire 1

^b^In singleton pregnancies with available data from the food frequency questionnaire (*n* = 83,721)

^c^In a subsample of *n* = 2001 with data on urinary iodine and creatinine in gestational week 18

^d^Use of a folic acid supplement within the interval from 4 weeks before to 8 weeks after conception from questionnaire 1

UIC measured in the subsample of 8-year-old MoBa-children (*n* = 279) indicated adequate iodine intake in the children (median UIC: 110 µg/L, IQR: 79, 155 µg/L). According to WHO criteria [21], median UIC > 100 µg/L is characterized as adequate in school-age children.

In our study sample, 6.9% of the 8 year olds were granted special education at school. According to the mother, 28% had suboptimal or low score on the mandatory mapping test in school in reading, and 18% had suboptimal or low score in mathematics. All outcome measures were correlated or partially overlapping. Maternally reported child language skills was correlated with reading skills (Spearman *r* = 0.26, *p* < 0.001) and writing skills (Spearman *r* = 0.29, *p* < 0.001). Among children receiving special educational services, 85% had suboptimal scores on the mapping tests in reading (81%) and/or mathematics (56%). We have previously reported significant associations of maternal iodine intake with child ADHD-symptoms reported in the same 8-year questionnaire [13]. ADHD-symptoms correlated with child language skills (Spearman *r* = 0.35, *p* < 0.001), and 31% of children with special education scored > 1.5 SD on ADHD symptoms. Venn diagrams further illustrating overlaps between outcomes are provided in Supplementary Figures S3–S6 (Online Resource 1).

We have previously reported that maternal iodine intake was associated with child language skills at age 3 years [12]. Eighty-one percent of the participants in the current sample were also included in the 3-year sample. Compared to those with normal language skills at 3 years (96%), children with mild to moderate language delay at 3 years (3.1%) scored on average 1.2 SD higher on the language score (CCC-S) at 8 years (95% CI 1.1, 1.3), and those with severe language delay at 3 years (0.7%) scored 2.8 SD higher (95% CI 2.3, 3.2). Higher scores at 8 years indicated poorer language skills.

### Iodine from food and outcomes

**Fig. 2:**
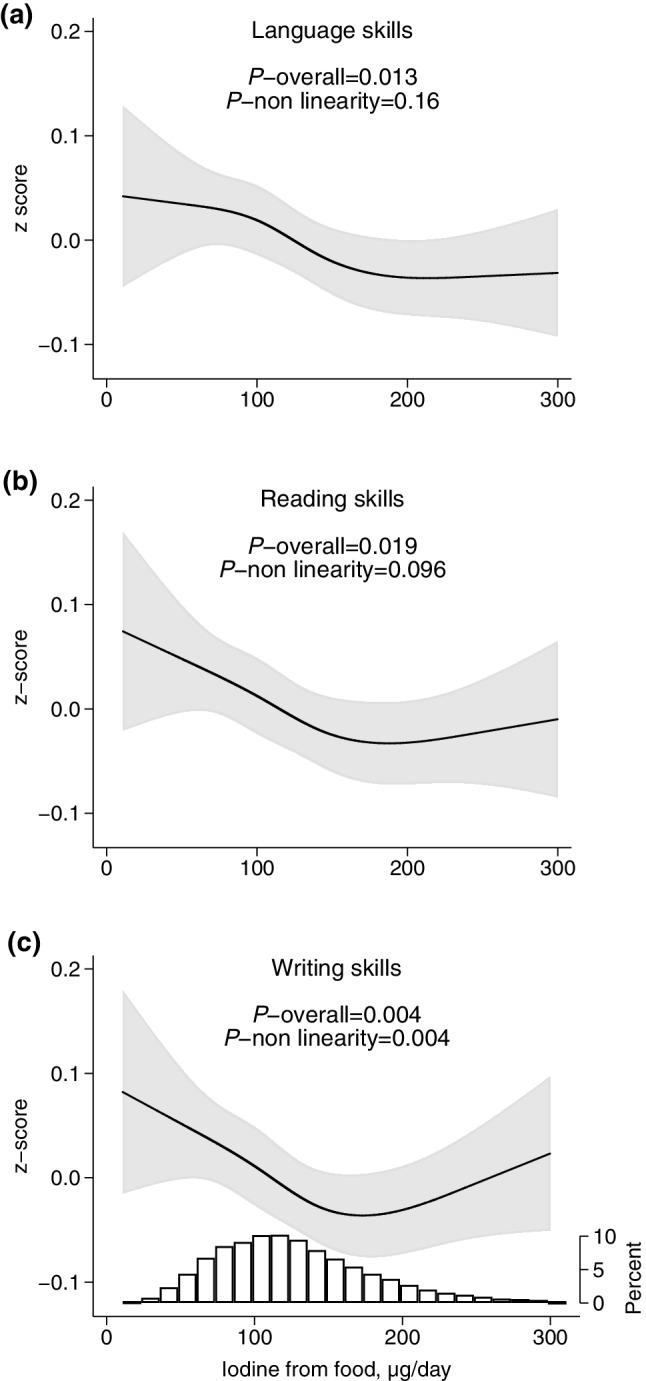
Maternal iodine intake from food (in non-supplement users) and child skills in language (**a**, *n* = 24,643), reading (**b**, *n* = 19,492), and writing (**c**, *n* = 19,483), adjusted models. Higher *z* scores indicate poorer skills. The histogram in **c** represents the distribution of iodine intake. For crude models, see Supplementary Figure S7 (Online Resource 1)

**Fig. 3:**
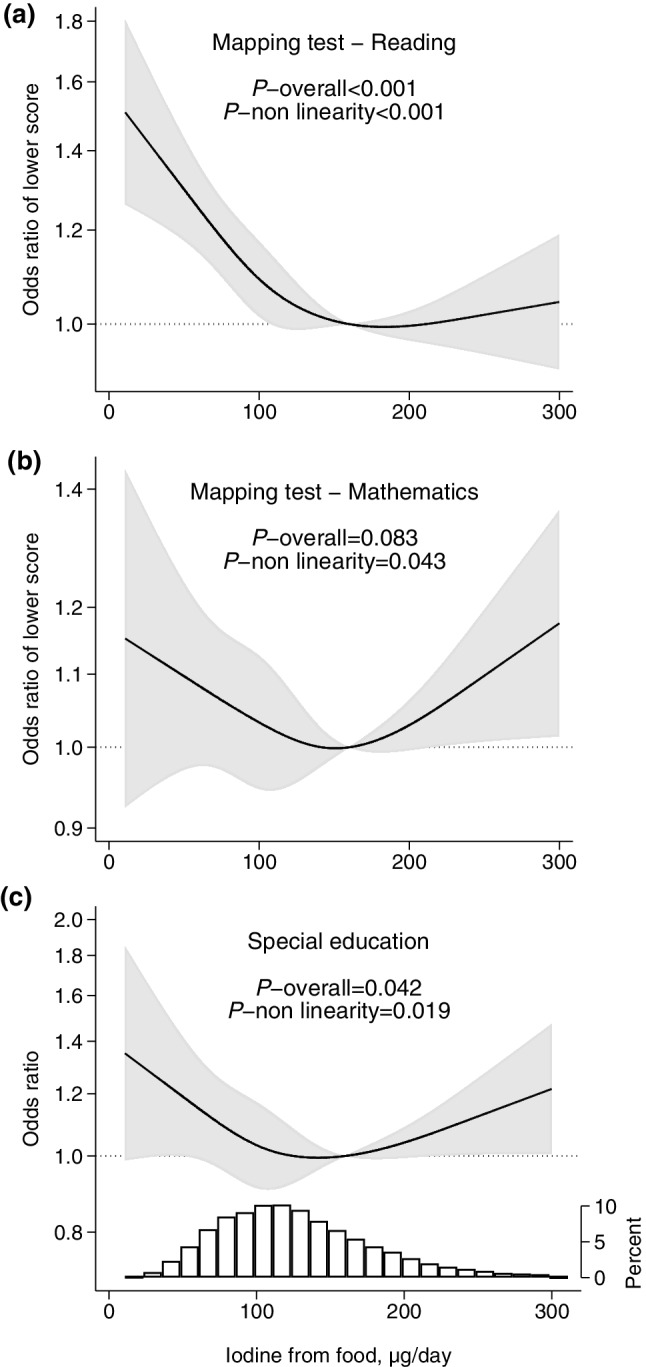
Maternal iodine intake from food (in non-supplement users) and child school outcomes at age 8 years, adjusted models. Higher odds ratio indicate poorer test results (**a, b**) and increased likelihood of receiving special educational services (**c**). The reference level (OR = 1) was set to 160 µg/day. Sample size was *n* = 24,309 for mapping tests in reading (**a**), *n* = 23,527 for mathematics (**b**), *n* = 24,806 for special education (**c**). The histogram in **c** represents the distribution of iodine intake. For crude models, see Supplementary Figure S8 (Online Resource 1)

Low maternal iodine intake from food was associated with the child having poorer skills in language, reading, and writing, and increased likelihood of special education (Figs. 2, 3). There was also a tendency for a lower performance in mathematics (Fig. 3b), but the association was not statistically significant (*p* = 0.083). Combined, the spline graphs indicated that habitual iodine intakes lower than about 150 µg/day was associated with lower child performance. Tabular results of the estimated associations are provided in Table 2.

**Table 2:** Iodine intake from food in pregnancy and language and learning at age 8 years in non-supplement users, adjusted models

Iodine intake (µg/day)	Language skills^a^	Reading skills^a^	Writing skills^a^	Mapping test Reading^b^	Mapping test Mathematics^b^	Special education
	*n*	*n*	*n*	*n*	*n*	*n*/*N* (%)
	24,643	19,492	19,483	24,309	23,527	1756/24,806 (7.1%)
	Std. beta (95% CI)	Std. beta (95% CI)	Std. beta (95% CI)	OR (95% CI)	OR (95% CI)	OR (95% CI)
25	0.07 (0.00, 0.13)	0.09 (0.02, 0.17)	0.11 (0.03, 0.18)	1.43 (1.23, 1.66)	1.13 (0.94, 1.36)	1.29 (1.00, 1.67)
50	0.06 (0.01, 0.11)	0.08 (0.03, 0.13)	0.09 (0.03, 0.14)	1.30 (1.18, 1.43)	1.10 (0.97, 1.24)	1.19 (1.00, 1.42)
75	0.06 (0.02, 0.09)	0.06 (0.02, 0.10)	0.07 (0.03, 0.11)	1.18 (1.10, 1.28)	1.06 (0.97, 1.16)	1.10 (0.96, 1.25)
100	0.04 (0.01, 0.08)	0.04 (0.00, 0.08)	0.05 (0.01, 0.08)	1.09 (1.01, 1.17)	1.03 (0.95, 1.13)	1.03 (0.91, 1.17)
125	0.03 (0.01, 0.05)	0.02 (0.00, 0.04)	0.02 (0.00, 0.05)	1.04 (0.99, 1.08)	1.01 (0.96, 1.07)	1.00 (0.92, 1.08)
160 (ref)	0	0	0	1	1	1
200	− 0.01 (− 0.02, 0.00)	0.00 (− 0.02, 0.01)	0.00 (− 0.01, 0.02)	1.00 (0.97, 1.03)	1.03 (1.00, 1.06)	1.04 (0.99, 1.09)
225	− 0.01 (− 0.03, 0.01)	0.00 (− 0.03, 0.03)	0.02 (− 0.01, 0.04)	1.01 (0.96, 1.06)	1.06 (1.00, 1.12)	1.08 (1.00, 1.16)
250	− 0.01 (− 0.04, 0.03)	0.01 (− 0.04, 0.05)	0.03 (− 0.01, 0.07)	1.02 (0.94, 1.10)	1.10 (1.01, 1.20)	1.12 (1.00, 1.26)
300	− 0.01 (− 0.07, 0.05)	0.02 (− 0.06, 0.10)	0.06 (− 0.01, 0.13)	1.04 (0.92, 1.19)	1.18 (1.01, 1.36)	1.22 (1.01, 1.47)
*p* overall	*p* = 0.013	*p* = 0.019	*p* = 0.004	*p* < 0.001	*p* = 0.083	*p* = 0.042
*p* non-linearity	*p* = 0.16	*p* = 0.096	*p* = 0.004	*p* < 0.001	*p* = 0.043	*p* = 0.019

Results are from multivariable regression analysis adjusting for confounders and for random effects of sibling clusters

^a^Standardized beta > 0 indicate poorer skills

^b^Odds ratio > 1 indicate increased risk of attaining poorer test results

Sensitivity analyses showed that associations were somewhat attenuated when adjusting for ADHD-symptoms at 8 years (Supplementary Figures S9 and S10, Online Resource 1), but remained largely the same (language skills and writing skills were no longer significant).

Maternal UIC in mid pregnancy (measured in a subgroup, *n* = 2001) was not significantly associated with any of the outcomes (Supplementary Figures S11 and S12, Online Resource 1), and adjustment for urinary creatinine to adjust for hydration level did not change the results (results not shown).

### Iodine from supplements

There was no evidence of any associations between maternal use of iodine-containing supplements and child outcomes (Supplementary Table 2, Online Resource 1). Interaction with iodine from food was tested, but there was no indication of interaction for any of the outcomes. Iodine intake from food did not differ by supplement use (including by timing of supplement use).

## Discussion

The main finding in this study is that low maternal habitual iodine intake (i.e., lower than ~ 150 µg/day from food) was associated with poorer maternally reported child performance, i.e., the child having poorer skills in language, reading, and writing, and also increased likelihood of the child receiving special education services. This finding is alarming and corroborate the previous findings in MoBa of increased risk of maternally reported language delay at child age 3 years [12]. Further, we did not detect any association between maternal iodine supplement use and child outcomes.

### Iodine intake from food and outcomes

Our findings suggest that impairments associated with mild-to-moderate ID in pregnancy persist although iodine intake is adequate in childhood (indicated by the subgroup median child UIC > 100 µg/L). Experimental animal studies have demonstrated that mild gestational ID can cause permanent neurological alterations in the developing brain [1]. Our findings are also supported by an observational study in Australia (*n* = 228, median gestational UIC: 81 µg/L) where children of mothers with gestational UIC < 150 µg/L had poorer educational outcomes (spelling, grammar and English-literacy) at age 9 years than peers whose mothers did not have mild or moderate gestational ID (i.e., UIC ≥ 150 µg/L), even though the children were iodine sufficient in childhood [15]. A follow-up study demonstrated that the reduced educational performance persisted also at 15 years of age [16]. Bath et al. found that children of mothers with a suboptimal UIC in pregnancy (< 150 µg/g creatinine) had a lower IQ and reading ability at child age 8–9 years in a UK cohort (*n* = 1040, median gestational UIC: 91 µg/L) [14]. However, limitations of these studies were that spot-UIC was the only measure of maternal iodine intake, and that the exposure was not modelled continuously. As a consequence, optimal intake was not explored and many participants may have been misclassified as deficient/not deficient.

We included a spot-UIC in mid pregnancy as an alternative measure of habitual iodine intake since data on maternal UIC was available for *n* = 2001 pregnant women. The null findings for UIC and outcomes (Supplementary Figures S11–S12, Online Resource 1) did not support our results that maternal habitual iodine intake was associated with the outcomes, but the seemingly conflicting results might have several explanations. Firstly, a single spot-UIC is a poor measure of habitual iodine intake at an individual level [40]. König et al. found that a minimum of ten repeated spot-UICs are needed to reliably estimate individual iodine intake [41]. Secondly, the subsample with UIC measurements was not a random sample of MoBa participants. They were selected from highly dedicated participants with complete datasets of questionnaires and biological samples up to child age 3 years, and the sample did not include children with language difficulties, or those with suspected or diagnosed autism. This reduces the power to identify potential associations with language and learning outcomes. In fact, we found that in this subsample, association curves between calculated iodine intake by the FFQ and outcomes were different from the total sample, and were more in agreement with the UIC findings (Supplementary Figures S11–S12, Online Resource 1).

We have previously reported that low maternal iodine intake was associated with child ADHD symptoms in the same population of 8-years-old MoBa-children [13]. In sensitivity analyses, we found that adjusting for maternally reported ADHD symptoms at child age 8 years did not change the results markedly (Supplementary Figures S9–S10, Online Resource 1), demonstrating that the associations between iodine intake with child language and learning were partly independent of score on ADHD-symptoms.

### Iodine intake from supplements

We have previously reported that women in MoBa who initiated use of iodine-containing supplements in the first trimester gave birth to children with more behaviour problems at age 3 [12] and 8 years [13] and increased risk of ADHD diagnosis by age 6–14 years [13]. However, iodine supplement use was not associated with child language- or motor skills at 3 years [12]. In the present study, there was no significant association between maternal use of iodine-containing supplements and child language and learning at 8 years. Summarized, results from MoBa are consistent in demonstrating no beneficial effects of iodine supplement use in pregnancy, but inconclusive as to whether it can be potentially harmful. Initiating use in pregnancy might be too late to compensate for a depleted maternal iodine store. The “jury is still out” on this question for areas of mild-to-moderate ID as recently concluded in a Cochrane review [22]. Nevertheless, the optimal strategy to prevent impairments caused by ID is to secure an adequate iodine status well before conception for all women of childbearing age [42].

### Strengths and limitations

Strengths of this study include the large size, prospective- and population-based design, and the substantial data collection allowing to control for a range of confounders. Additionally, in Norway there is a large variation in habitual iodine intake since only milk and salt-water fish are important food sources of iodine in the Norwegian diet [43]. Consequently, we could study a wide range of iodine intake with flexible modelling techniques and explore potential non-linear associations. The multiple outcomes on child language and learning included results on mapping tests and whether the child was granted special education. These outcomes are more objective than the subjective evaluation of the child’s language, reading and writing skills evaluated and reported by the mother which may include substantial measurement error. Child language skills were also measured in the 3-year questionnaire [12], and ideally, we would have modelled this repeated outcome longitudinally. Unfortunately this was not feasible since the instruments used were not the same at ages 3 and 8, and consequently did not measure the exact same dimensions.

Calculating iodine intake by an FFQ is inaccurate, both due to a natural variation of iodine content of food, limited quality of the data on food iodine content, the FFQ not covering all possible food and drink alternatives, deliberate or undeliberate misreporting, and an FFQ being a survey format requiring some literacy skills to complete correctly. Nevertheless, the previous validation study showed acceptable agreement between the MoBa FFQ calculations, 24 h UIC, and a 4 days weighed-food dairy [35]. Additionally, since there are few food sources of iodine in Norway, a person’s iodine intake is largely dependent on individual food choices and can be easier to estimate than many other nutrients. In the FFQ, women were asked to report their habitual food intake since the beginning of pregnancy. The results of this study might reflect not only iodine intake during pregnancy but also habitual iodine intake prior to pregnancy and thus maternal iodine stores.

The low participation rate in MoBa (41%) is a concern. Women participating in MoBa are more often nulliparous, non-smokers, older and better educated than non-participants [44]. These are attributes generally associated with healthier diet and lifestyles, and yet both UIC and calculated iodine intake confirmed insufficient iodine intake, and there is little reason to expect that non-participants would have higher iodine intake than participants. Loss to follow-up is another concern, and only 35% of recruited women completed the 8-year questionnaire. Loss to follow-up is inevitable and commonly leads to selection bias and loss of statistical power [45]. In this study, iodine intake from food and iodine supplement use did not differ between responders and non-responders to the 8 years questionnaire. Still, we cannot exclude that loss to follow-up have influenced the results. Finally, as in all epidemiological studies, residual confounding might still be present due to the observational design, and thus we cannot make causal inference based on the results.

### Clinical relevance and implications

Although the estimated effects of mild-to-moderate ID in this study are small, the high prevalence of insufficient maternal iodine intake worldwide indicates that a substantial proportion of children might not reach their full developmental potential as a consequence of maternal mild-to-moderate ID. In MoBa, 69% had a calculated iodine intake from food < 150 µg/day which was associated with poorer child outcomes. In Europe, two-thirds of countries that have assessed iodine nutrition in pregnant women have reported inadequate intakes [46]. The cost of insufficient iodine nutrition is high both at the individual and societal level [18], whereas the cost of salt iodization, the primary strategy recommended by the WHO, is low [42]. Our findings support the current recommendations by the WHO that it is of vital importance to secure sufficient iodine intake of > 150 µg/day in women of childbearing age [21].

In Norway, the health authorities have now, since 2017, initiated several actions to prevent ID. The national guidelines for a healthy diet now specifies that three portions of dairy products per day can contribute to secure an adequate intake of iodine and calcium. They recommend iodine supplements for women of childbearing age, pregnant, and lactating women who have a low milk/yoghurt intake. In addition, they are currently evaluating strategies to increase iodine intake by salt iodization.

## Conclusions

A low habitual iodine intake in pregnant women, i.e., lower than the recommended daily intake for non-pregnant women, was associated with mothers reporting slightly poorer child language, school performance, and increased likelihood of special educational services at child age 8 years. We found no indications of benefits or harm of using iodine-containing supplements in pregnancy. Initiating use in pregnancy might be too late.

## Electronic supplementary material

Below is the link to the electronic supplementary material.

Supplementary material 1 (DOCX 901 KB)

## Abbreviations

EAR: Estimated average requirement
FFQ: Food frequency questionnaire
GW: Gestational week
ID: Iodine deficiency
MoBa: The Norwegian Mother and Child Cohort Study
UIC: Urinary iodine concentration
