# Supplementary material for: Language delay and poorer school performance in children of mothers with inadequate iodine intake in pregnancy: results from follow-up at 8 years in the Norwegian Mother and Child Cohort Study
Source: Eur J Nutr. 2018 Nov 12;58(8):3047–58. doi: 10.1007/s00394-018-1850-7 (PMC6842354; doi:10.1007/s00394-018-1850-7)
Supplement: Supplementary file 1 — Supplementary material 1 (DOCX 901 KB) [file 394_2018_1850_MOESM1_ESM.docx]

# Supplementary material to: Abel MH^123^ , Brandlistuen RE^1^, Caspersen IH^1^, Aase H^1^, Torheim LE^2^, Meltzer HM^1^, Brantsæter AL^1*^ Language delay and poorer school performance in children of mothers with inadequate iodine intake in pregnancy - results from follow up at 8 years in the Norwegian Mother and Child Cohort Study. European Journal of Nutrition (2018)

^1^ Norwegian Institute of Public Health, Oslo, Norway
^2^ Oslo Metropolitan University, Norway
^3^ TINE SA, Oslo, Norway
* Corresponding author: Anne Lise Brantsæter (e-mail: annelise.brantsaeter@fhi.no)

**Supplementary Figure S1** Conceptual model (simplified directed acyclic diagram (DAG))


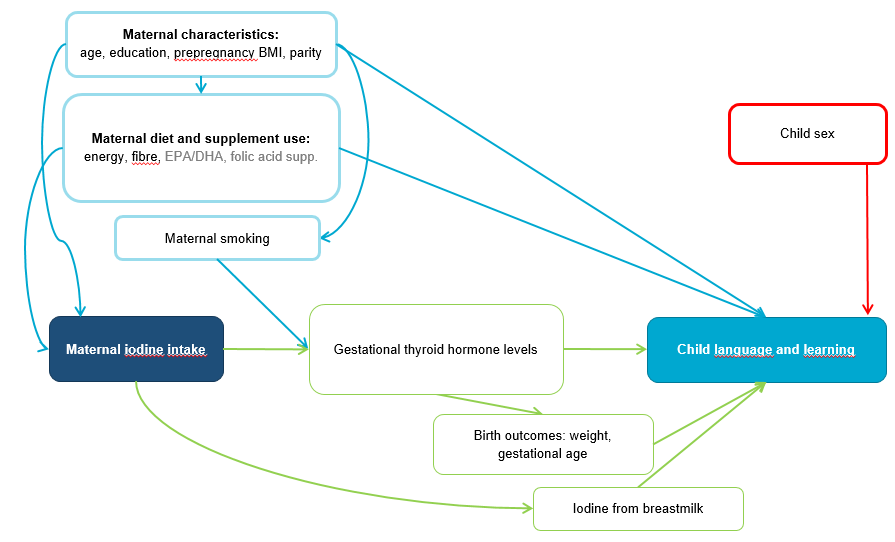


Maternal history of read/write difficulties

Association between maternal iodine intake and child language and learning. Potential causal pathways are illustrated in green and confounding pathways in blue. Child sex and maternal history of reading and writing difficulties are important determinants of the outcome marked in red. Intake of the n-3 fatty acids EPA and DHA and reported use of folic acid supplements were only included as confounders when iodine from supplements was the exposure.

### Supplementary Table S1 Iodine exposures by characteristics of the study population (n=39,471 mother-child pairs)

|  | **Study population** | **Iodine from food** | **Iodine supplement** | **UIC ^a^** |
| --- | --- | --- | --- | --- |
|  |  | median (IQR) | **G**W 0-22 | median (IQR) |
|  |  | µg/day | **%** | µg/L |
| Study sample, n (%) | 39,471 (100) | 122 (89, 161) | 37 | 67 (35, 115) |
| Maternal age at delivery, mean (SD), years | 30.6 (4.4) |  |  |  |
| <25 | 8.0 | 122 (85, 169) | 37 | 66 (39, 107) |
| 25-34 | 73 | 121 (89, 160) | 37 | 66 (35, 114) |
| ≥35 | 19 | 123 (91, 160) | 37 | 71 (33, 122) |
| Pre-pregnancy BMI, mean (SD), kg/m^2^ | 23.8 (4.1) |  |  |  |
| <18.5 | 2.7 | 122 (89, 161) | 41 | 71 (31, 117) |
| 18.5-24.9 | 66 | 122 (90, 160) | 38 | 66 (34, 113) |
| 25-30 | 21 | 121 (87, 162) | 35 | 70 (33, 118) |
| >30 | 8.0 | 118 (84, 160) | 36 | 76 (45, 122) |
| Missing | 2.4 | 125 (91, 163) | 34 | 61 (35, 123) |
| Parity, % |  |  |  |  |
| 0 | 47 | 118 (87, 158) | 43 | 65 (34, 114) |
| 1 | 35 | 122 (90, 160) | 34 | 70 (35, 118) |
| 2 or more | 17 | 128 (95, 168) | 29 | 68 (40, 102) |
| Missing | 0.3 | 117 (82, 159) | 30 | - |
| Maternal education, % |  |  |  |  |
| ≤12 y | 24 | 122 (87, 166) | 34 | 69 (40, 116) |
| 13-16 y | 45 | 122 (90, 161) | 38 | 67 (33, 113) |
| >16 y | 29 | 120 (91, 155) | 40 | 67 (33, 119) |
| Other/missing | 2.1 | 117 (85, 157) | 36 | 66 (42, 112) |
| Married/cohabitant, % |  |  |  |  |
| Yes | 96.8 | 122 (90, 160) | 37 | 67 (35, 115) |
| No | 2.9 | 120 (87, 165) | 39 | 79 (35, 109) |
| Missing | 0.3 | 118 (82, 159) | 30 | - |
| Smoking in pregnancy, % |  |  |  |  |
| Occasionally | 14 | 120 (87, 161) | 38 | 65 (32, 109) |
| Daily | 3.5 | 120 (88, 166) | 32 | 65 (32, 99) |
| Chronic illness, % | 9.9 | 116 (83, 156) | 41 | 65 (33, 108) |
| Household income, % |  |  |  |  |
| Low | 24 | 124 (91, 167) | 35 | 69 (38, 110) |
| Medium | 42 | 123 (90, 162) | 36 | 66 (34, 119) |
| High | 32 | 118 (88, 153) | 40 | 69 (34, 114) |
| Missing | 2.3 | 126 (92, 170) | 34 | 64 (44, 113) |
| Bilingual parent(s), % | 9.7 | 119 (87, 155) | 42 | 60 (34, 114) |
| Maternal history of reading/writing difficulties (%) | 5.6 | 118 (85, 161) | 38 | 74 (45, 133) |
| Child sex boy (%) | 50.9 | 122 (90, 161) | 37 | 66 (34, 116) |
| Iodine supplement in pregnancy, % |  |  |  |  |
| No | 63 | 122 (90, 161) | 0 | 59 (32, 101) |
| Yes | 37 | 121 (89, 160) | 100 | 83 (43, 138) |
| Current user in GW 17-20 | 18 | 121 (89, 160) | 100 | 95 (50, 152) |
| Folic acid supplement (%) | 75 | 121 (89, 159) | 42 | 70 (36, 120) |
| Omega 3 supplement (%) | 80 | 123 (91, 161) | 42 | 69 (35, 119) |
| Maternal energy intake, median (IQR), MJ | 9.3 (7.9, 11.0) |  |  |  |
| Iodine from food, median (IQR), µg/day | 122 (89, 161) |  |  |  |
| <75 | 15 | 61 (51, 68) | 37 | 41 (24, 72) ^b^ |
| 75-149.9 | 54 | 112 (95, 129) | 37 | 59 (31, 100) ^b^ |
| ≥150 | 30 | 186 (165, 220) | 37 | 70 (39, 116) ^b^ |
| Urinary creatinine, median (90% range) g/L**^a^** | 0.74 (0.17, 1.90) |  |  |  |
| UIC, median (IQR), µg/g creatinine**^a^** | 91 (63, 139) |  |  |  |

^a^ Urinary iodine concentration (UIC) was measured in a subsample of n=2001 pregnant women in mean gestational week 18.5 (SD: 1.3).
^b^ In non-users of iodine-containing supplements (n=1208)


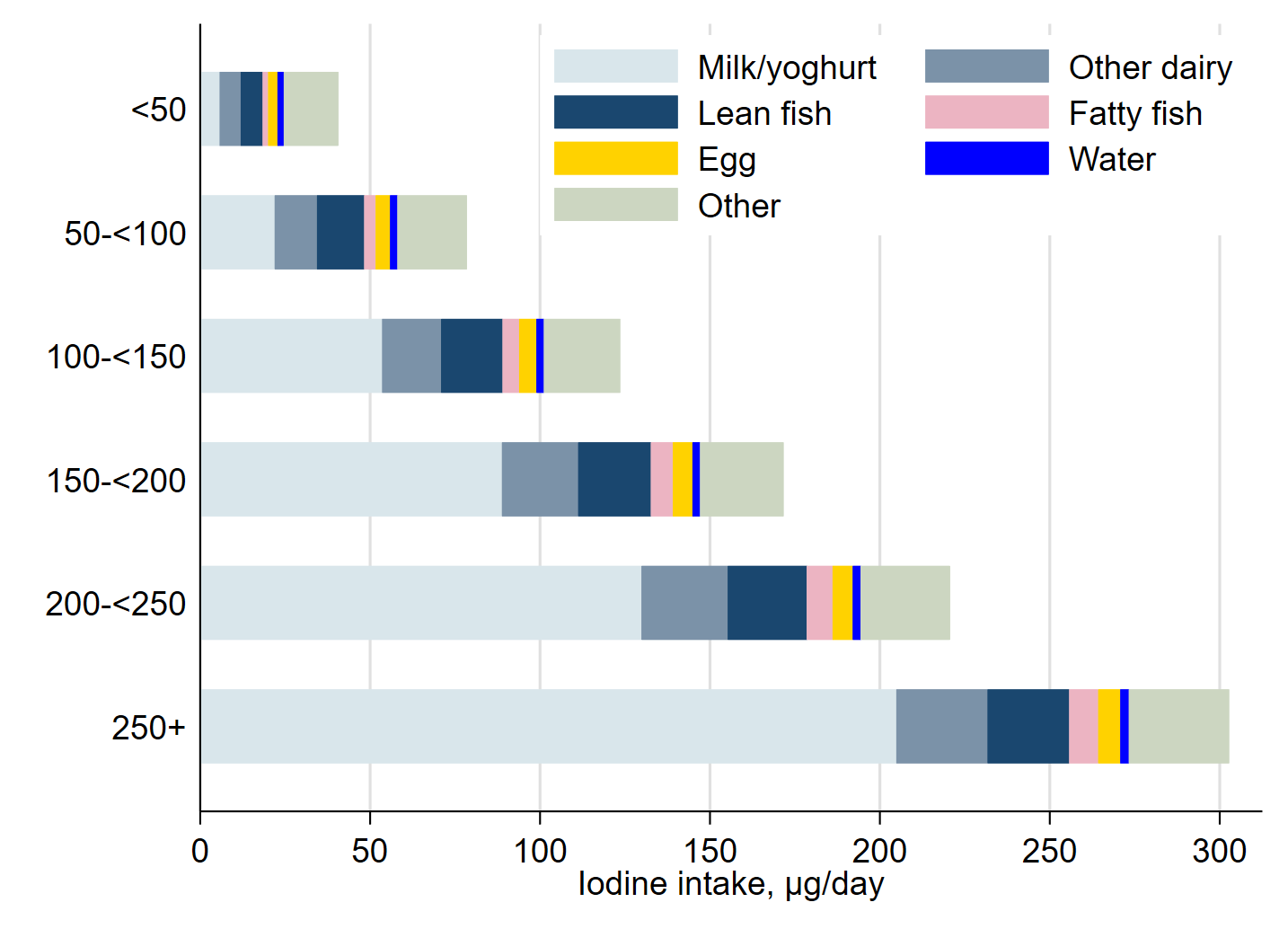


**Supplementary Figure S2** The mean contribution to iodine intake by different food sources by categories of total iodine intake from food (n=29,471). The vertical axis show the iodine intake intervals (in µg/day) for the categories.


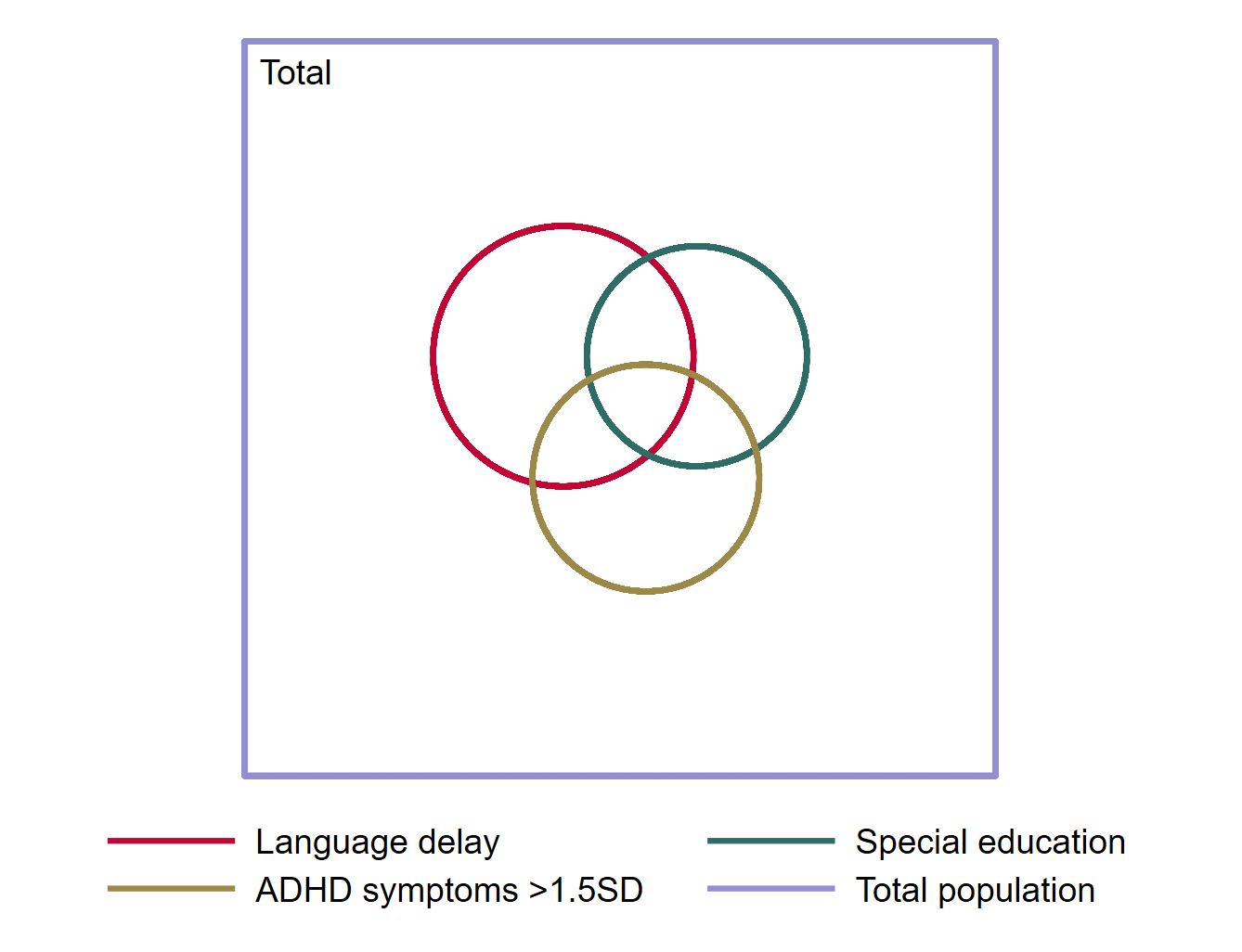


**Supplementary Figure S3** Venn diagram illustrating the overlap of children scoring high on ADHD symptoms (18 items from the ADHD rating scale), children that were granted special education in school, and children with language delay (scoring above the 90^th^ percentile on the Children’s Communication Checklist, or mother reported language delay as a current health problem).


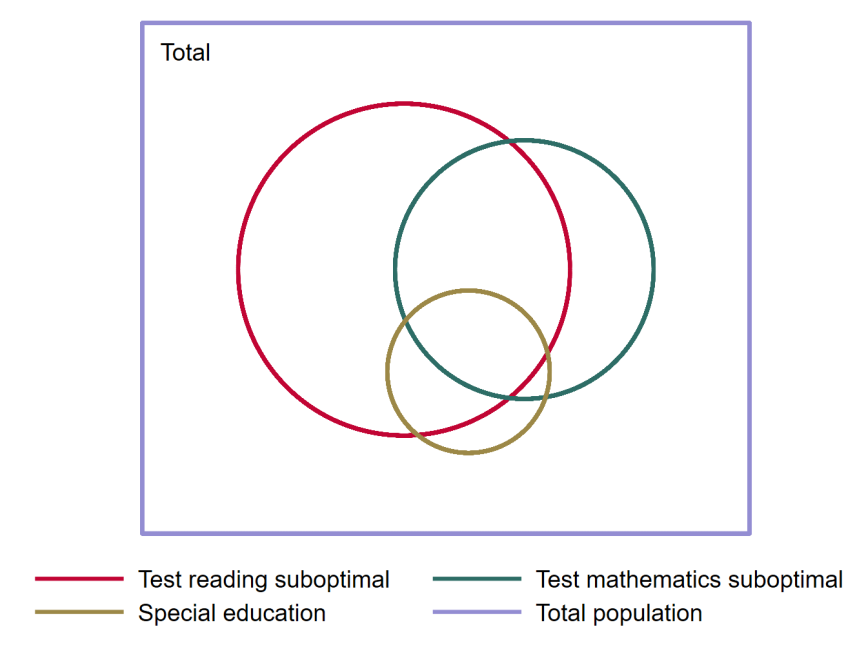


**Supplementary Figure S4** Venn diagram illustrating the overlap of children granted special education in school, and children with suboptimal results on the mandatory mapping tests in reading and mathematics.


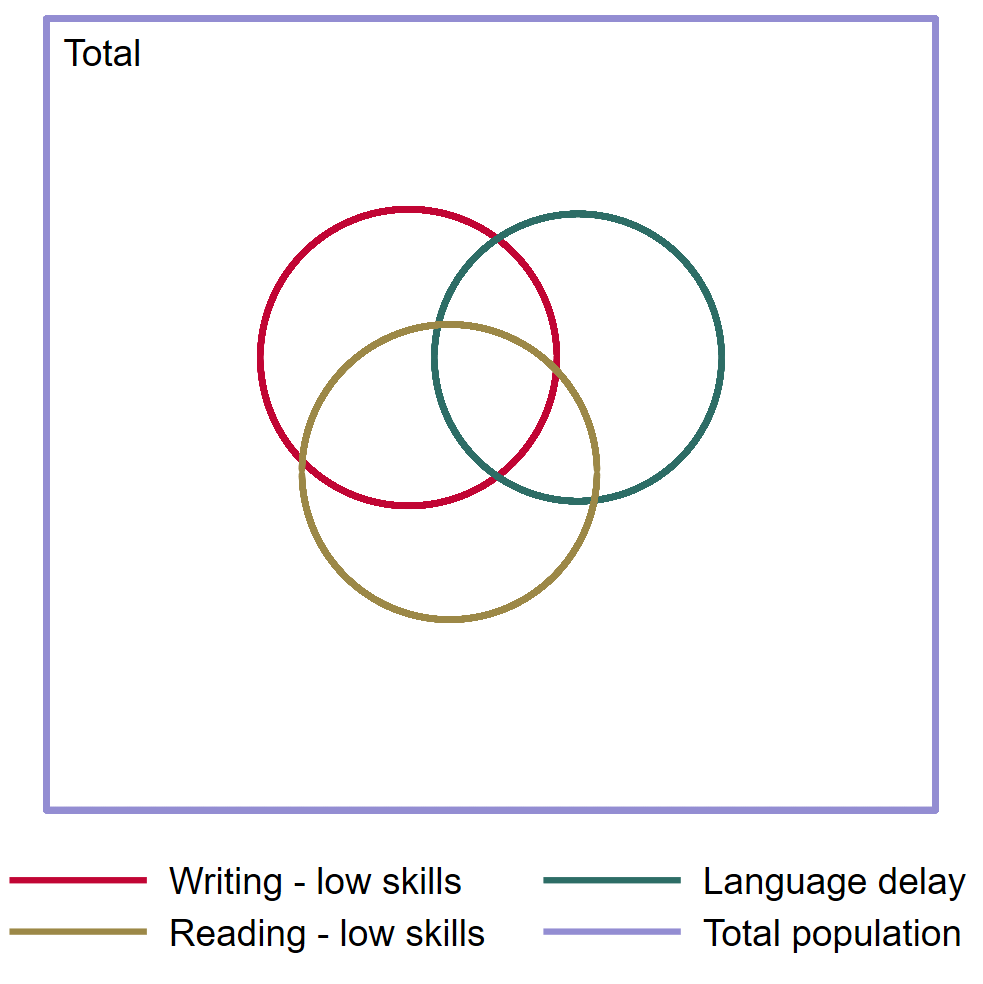


**Supplementary Figure S5** Venn diagram illustrating the overlap of children with scores >90^th^ percentile on maternally rated reading and writing skills (higher score for lower skills), and language delay (scoring above the 90^th^ percentile on the Children’s Communication Checklist, or mother reported language delay as a current health problem).


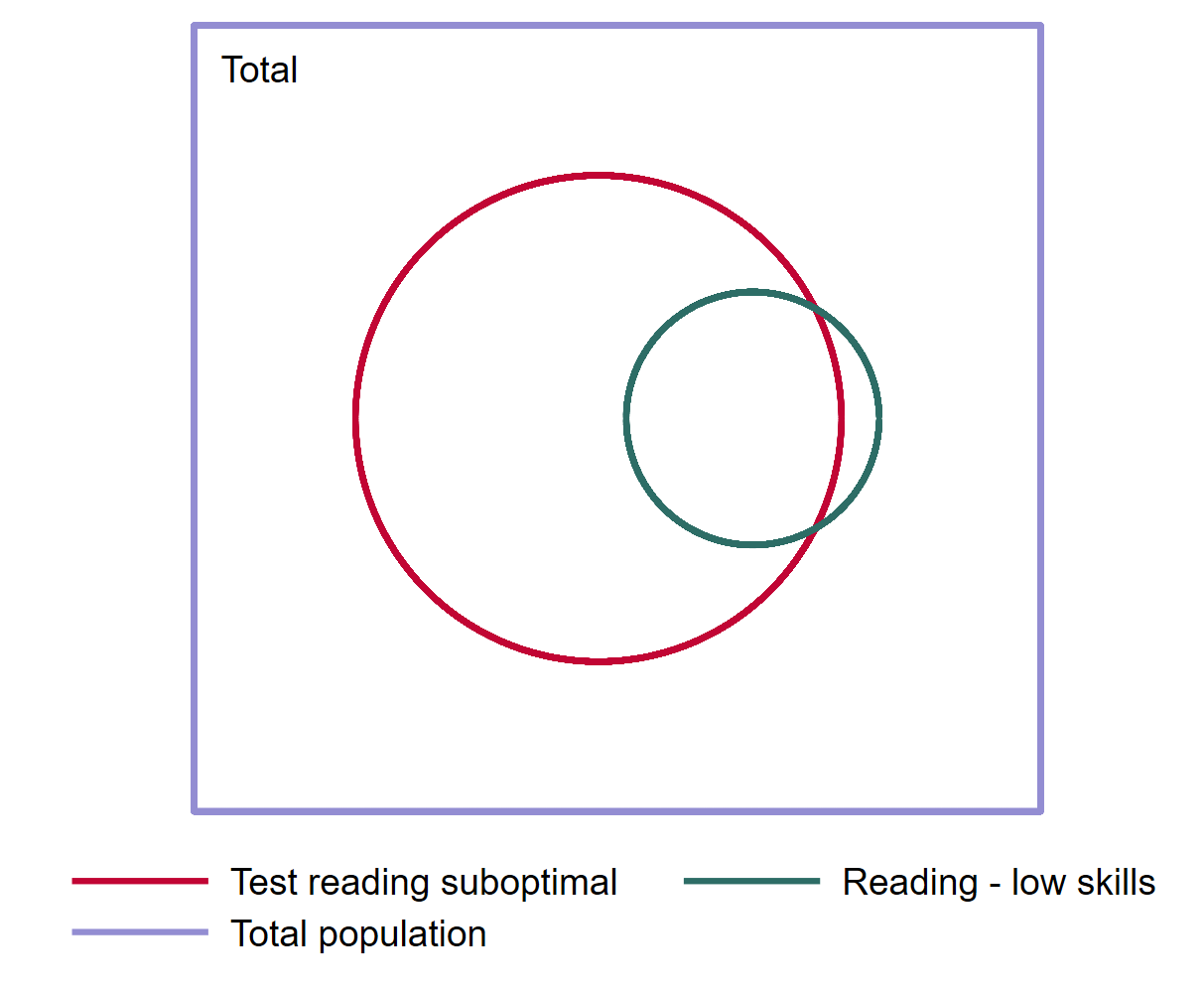


**Supplementary Figure S6** Venn diagram illustrating the overlap of children with suboptimal results on the mandatory mapping tests in reading, and children with scores >90^th^ percentile on maternally rated reading and writing skills (higher score for lower skills).


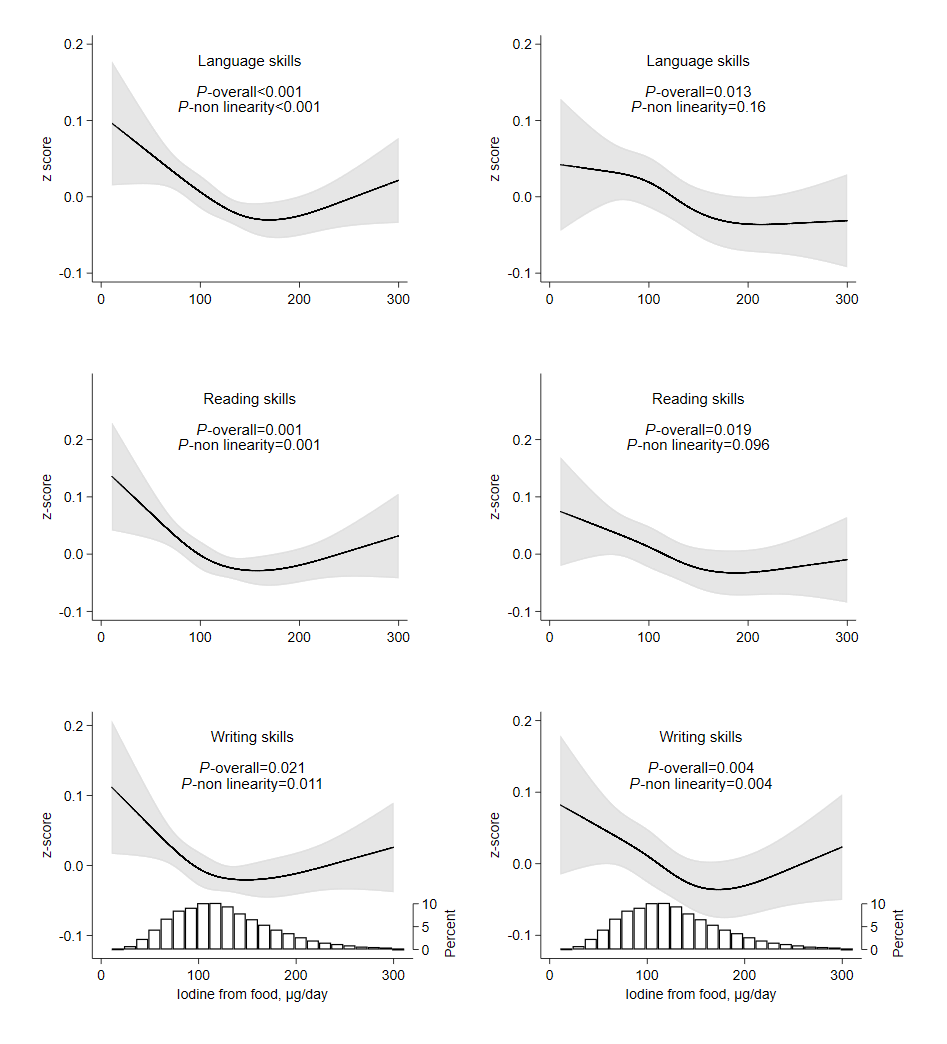


**Supplementary Figure S7** **Crude (left) and adjusted (right) associations** of maternal iodine intake from food (in non-supplement users) and child language (n=24,643), reading (n=19,492), and writing skills (n=19,483) at age 8 years. Higher z-score indicate poorer skills. Associations were modelled flexibly (restricted cubic splines, four knots) and estimated by generalized linear regression. All models were adjusted for energy intake and random effects of sibling clusters. Adjusted models were additionally adjusted for maternal age, education, parity, pre-pregnancy BMI, fibre intake, smoking in pregnancy, child sex, bilingual parent(s) (for the language outcome), and maternal history of reading/writing difficulty (for read/write scores). The histogram represents the distribution of iodine intake from food. Missing data on covariates were imputed (4.2% of participants had missing data for one or more covariate).


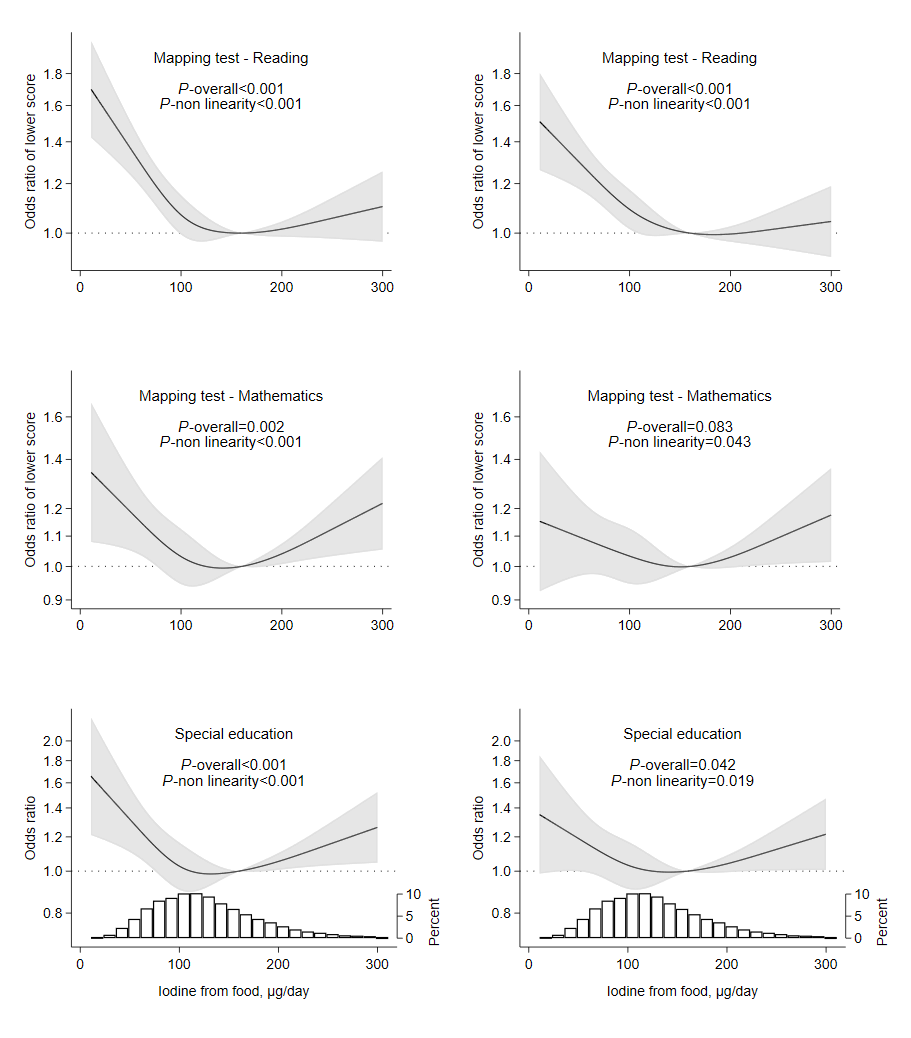


**Supplementary Figure S8** **Crude (left) and adjusted (right) associations** of maternal iodine intake from food (in non-supplement users) and school outcomes at age 8 years. Higher odds ratio indicate poorer test results/increased likelihood of receiving special educational services. The reference level (OR=1) was set to 160 µg/day, the estimated average requirement in pregnancy by the Institute of Medicine. Associations were modelled flexibly (restricted cubic splines, four knots) and estimated by ordered logistic regression for the mapping tests (n=24,309 for reading and n=23,527 for mathematics) and by logistic regression for special education (n=24,806). All models were adjusted for energy intake and random effects of sibling clusters. Adjusted models were additionally adjusted for maternal age, education, parity, pre-pregnancy BMI, fibre intake, and smoking in pregnancy. The histogram represents the distribution of iodine intake from food. Missing data on covariates were imputed (4.2% of participants had missing data for one or more covariate).


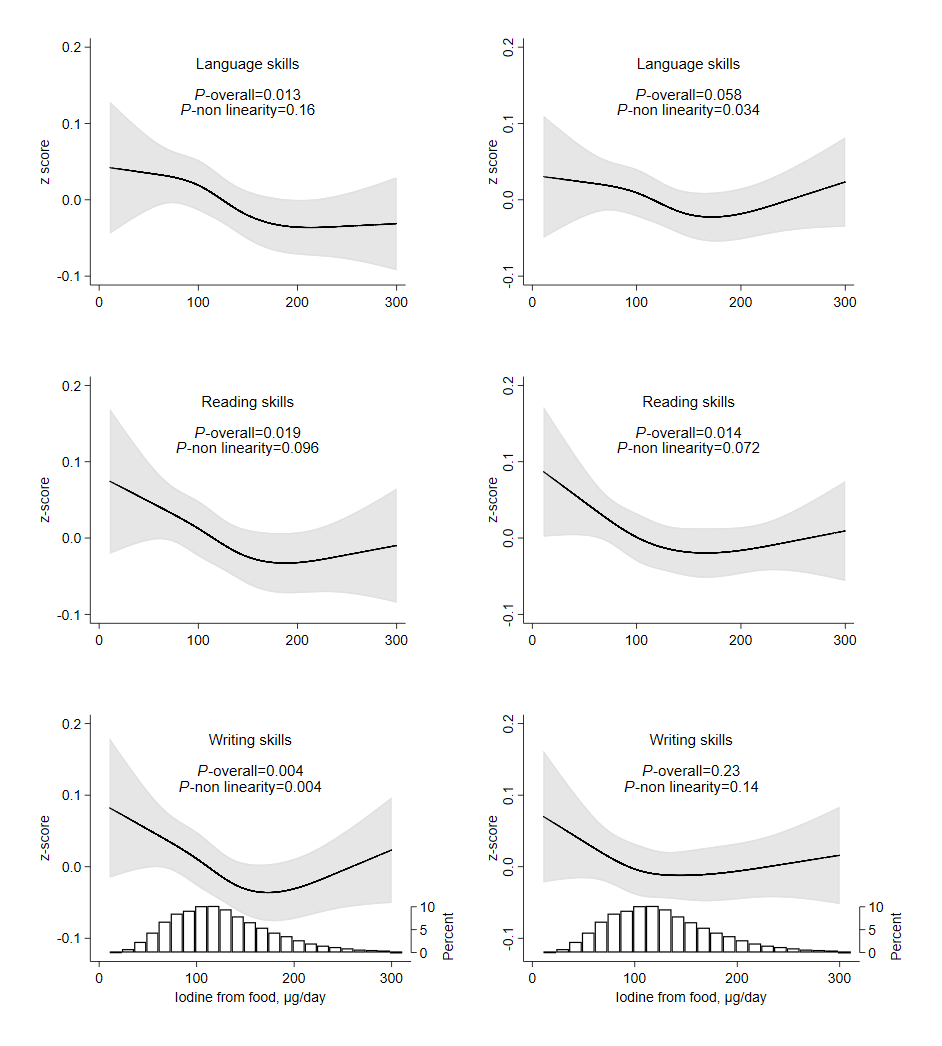


**Supplementary Figure S9** Adjusted models (same as in the manuscript) to the left and **models additionally adjusted for maternally reported ADHD symptoms at age 8 years to the right**. Associations of maternal iodine intake from food (in non-supplement users) and child language (n=24,643), reading (n=19,492), and writing skills (n=19,483) at age 8 years. Higher z-score indicate poorer skills. Associations were modelled flexibly (restricted cubic splines, four knots) and estimated by generalized linear regression. All models were adjusted for maternal age, education, parity, pre-pregnancy BMI, energy intake, fibre intake, smoking in pregnancy, child sex, bilingual parent(s) (for the language outcome), maternal history of reading/writing difficulty (for read/write scores), and for random effects of sibling clusters. The histogram represents the distribution of iodine intake from food. Missing data on covariates were imputed (4.2% of participants had missing data for one or more covariate).


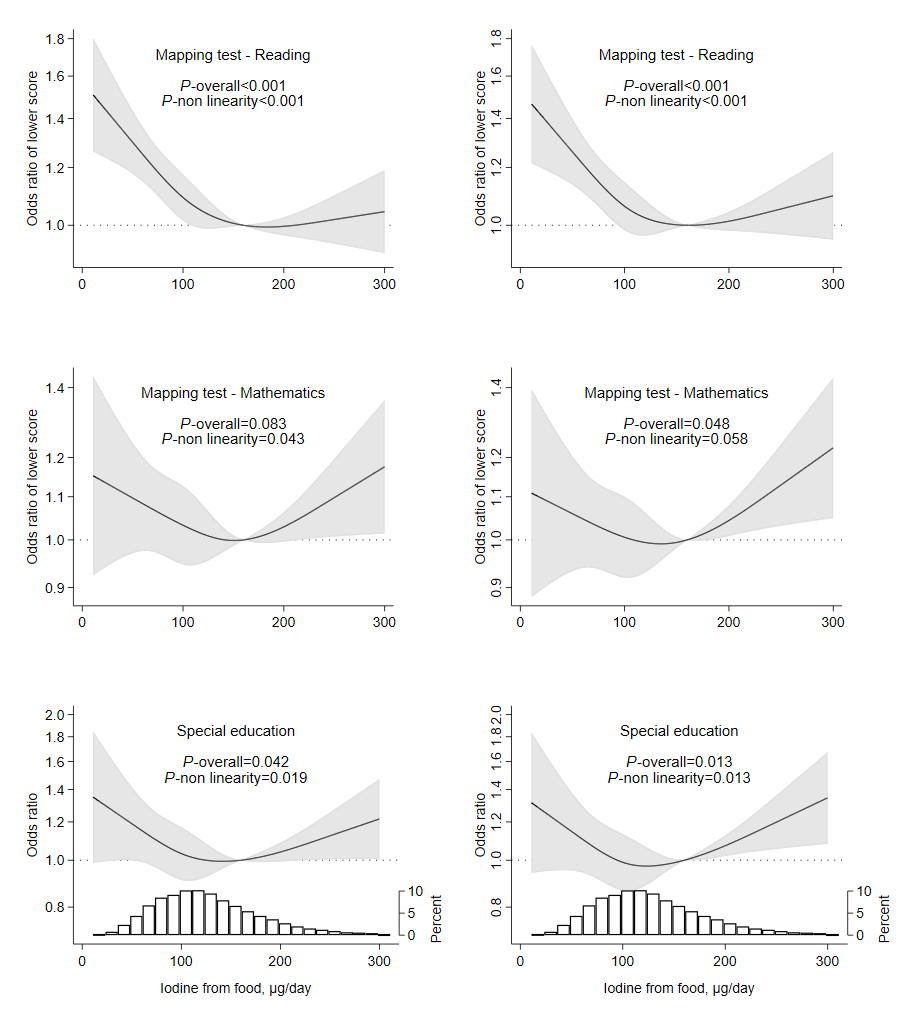


**Supplementary Figure S10** Associations of maternal iodine intake from food (in non-supplement users) and school outcomes at age 8 years. Adjusted models (same as in the manuscript) to the left and **models additionally adjusted for maternally reported ADHD symptoms at age 8 years to the right**. Higher odds ratio indicate poorer test results/increased likelihood of receiving special educational services. The reference level (OR=1) was set to 160 µg/day, the estimated average requirement in pregnancy by the Institute of Medicine. Associations were modelled flexibly (restricted cubic splines, four knots) and estimated by ordered logistic regression for the mapping tests (n=24,309 for reading and n=23,527 for mathematics) and by logistic regression for special education (n=24,806). Models were adjusted for maternal age, education, parity, pre-pregnancy BMI, energy intake, fibre intake, and smoking in pregnancy. The histogram represents the distribution of iodine intake from food. Missing data on covariates were imputed (4.2% of participants had missing data for one or more covariate).


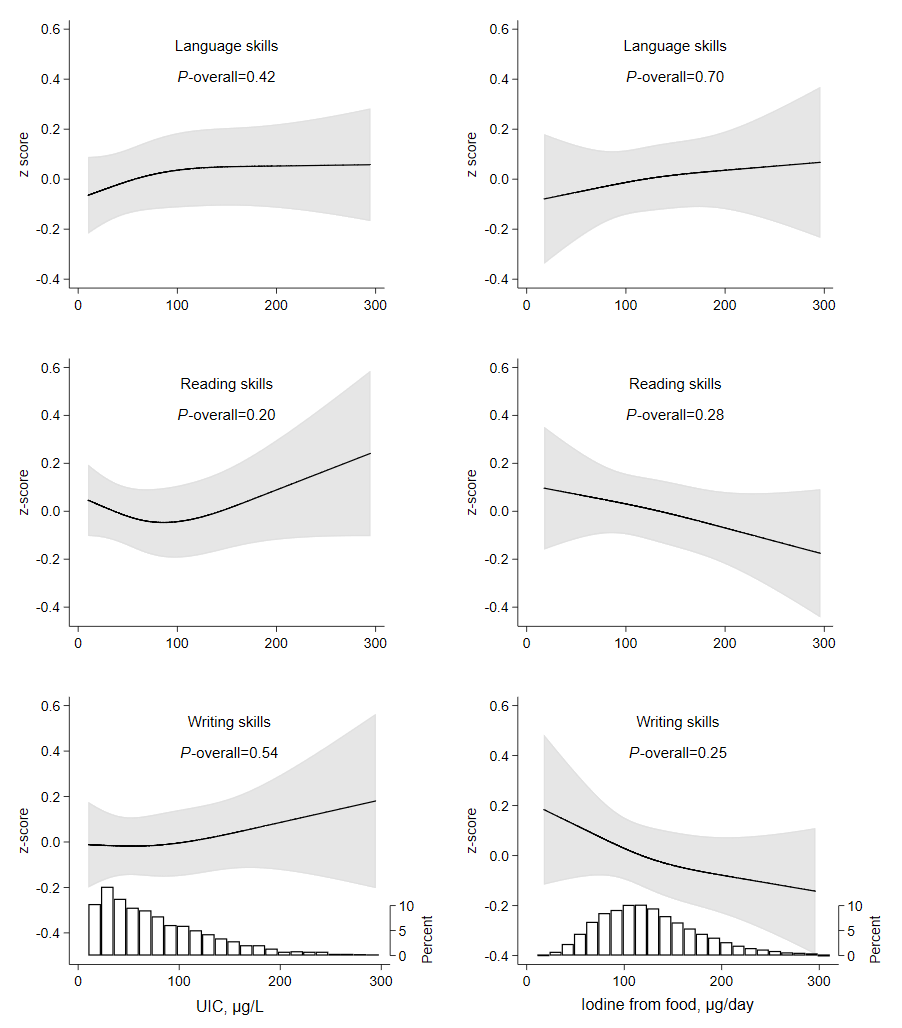


**Supplementary Figure S11** In subsample of non-users of iodine-containing supplements with available data on urinary iodine concentration (mean gestational week mean: 18.5, SD: 1.3): **Maternal urinary iodine concentration (left column), iodine intake from food by the food frequency questionnaire (right column) and maternally reported child skills** (language (n=1158), reading (n=1116), and writing (n=1117)). Higher z-score indicate poorer skills. Models were adjusted for maternal age, education, parity, pre-pregnancy BMI, energy intake, fibre intake, smoking in pregnancy, child sex, bilingual parent(s) (for the language outcome), maternal history of reading/writing difficulty (for read/write scores), and for random effects of sibling clusters. Associations were modelled for complete cases (no missing covariates). The histograms represent the distribution of UIC and of iodine intake from food. Results were similar when using UIC adjusted for hydration status (i.e. UIC in µg/g creatinine), and when also including the iodine supplement users (results not shown).


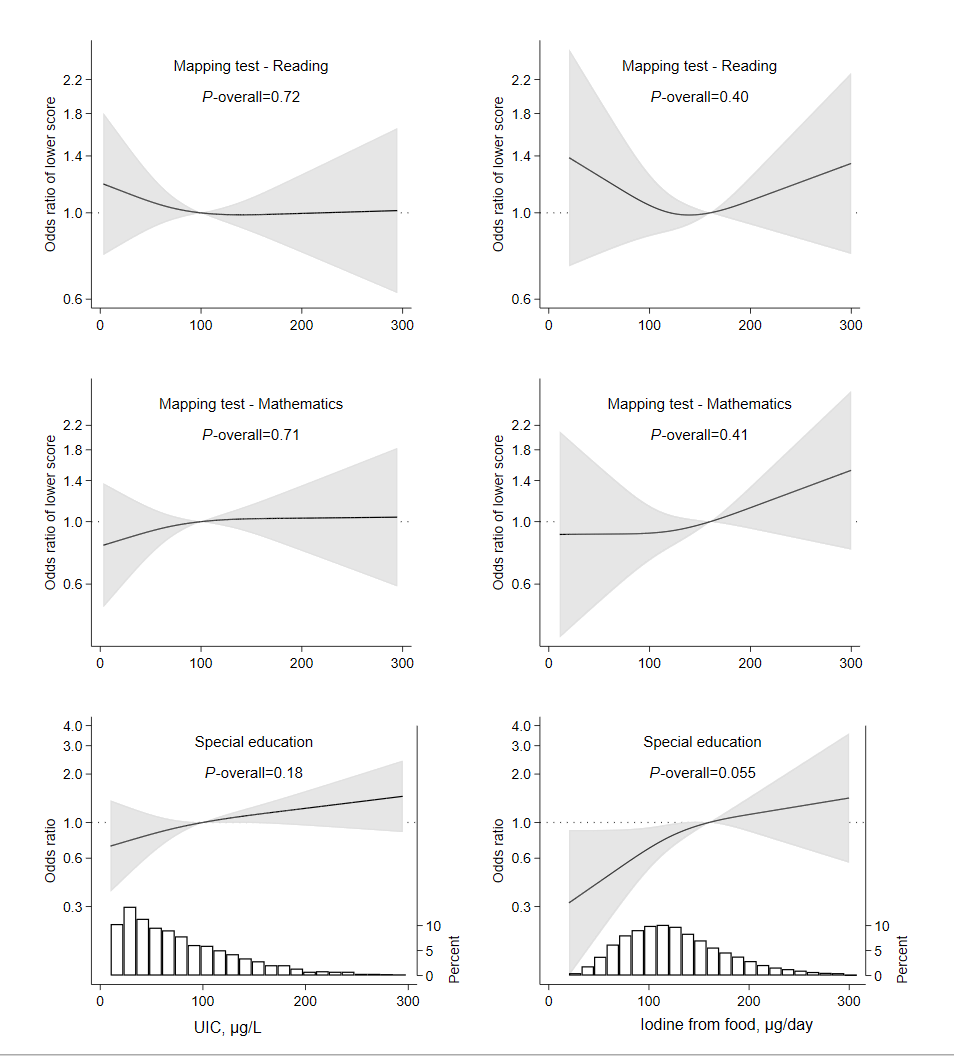


**Supplementary Figure S12** In a subsample of non-users of iodine-containing supplements with available data on urinary iodine concentration (mean gestational week mean: 18.5, SD: 1.3): **Maternal urinary iodine concentration (left column), iodine intake from food by the food frequency questionnaire (right column) and school outcomes** (reading (n=1145), mathematics (n=1110), and special education (n=1162)). Higher odds ratio indicate poorer test results/increased likelihood of receiving special educational services. The reference level (OR=1) was set to 160 µg/day, the estimated average requirement in pregnancy by the Institute of Medicine. Models were adjusted for maternal age, education, parity, pre-pregnancy BMI, energy intake, fibre intake, and smoking in pregnancy. Associations were modelled for complete cases (no missing covariates. The histograms represent the distribution of UIC and of iodine intake from food.). Results were similar when using UIC adjusted for hydration status (i.e. UIC in µg/g creatinine), and when also including the iodine supplement users (results not shown).

### Supplementary Table S2 Use of iodine-containing supplements in pregnancy and child language and learning at 8 years

|  | ***n*** | **Crude models^a^** | **Adjusted models^b^** |
| --- | --- | --- | --- |
|  |  |  |  |
| **Language skills** | 39,229 | Standardized beta (95% CI) | Standardized beta (95% CI) |
|  |  |  |  |
| Any iodine supplement use GW 0-22 | 14,586 | - 0.02 (-0.04, 0.00) | 0.00 (-0.02, 0.02) |
| First report of iodine supplement |  |  |  |
| Never (non-supplement user) | 24,643 | 0 (ref.) | 0 (ref.) |
| Before pregnancy^c^ | 4076 | -0.03 (-0.06, 0.00) | 0.00 (-0.03, 0.03) |
| GW 0-12 | 3521 | -0.04 (-0.07, 0.00) | 0.00 (-0.03, 0.03) |
| GW >12 | 2642 | -0.04 (-0.08, 0.00) | -0.02 (-0.06, 0.01) |
|  |  |  |  |
| **Reading skills** | 31,822 | Standardized beta (95% CI) | Standardized beta (95% CI) |
|  |  |  |  |
| Any iodine supplement use GW 0-22 | 12,330 | -0.03 (-0.05, 0.00) | - 0.01 (-0.03, 0.01) |
| First report of iodine supplement |  |  |  |
| Never (non-supplement user) | 19,492 | 0 (ref.) | 0 (ref.) |
| Before pregnancy^c^ | 3466 | -0.05 (-0.08, -0.01) | -0.02 (-0.05, 0.02) |
| GW 0-12 | 2976 | -0.01 (-0.05, 0.03) | 0.02 (-0.02, 0.06) |
| GW >12 | 2138 | -0.06 (-0.10, -0.02) | -0.04 (-0.08, 0.00) |
|  |  |  |  |
| **Writing skills** | 31,812 | Standardized beta (95% CI) | Standardized beta (95% CI) |
|  |  |  |  |
| Any iodine supplement use GW 0-22 | 12,329 | -0.04 (-0.06, -0.01) | - 0.01 (-0.04, 0.01) |
| First report of iodine supplement |  |  |  |
| Never (non-supplement user) | 19,483 | 0 (ref.) | 0 (ref.) |
| Before pregnancy^c^ | 3467 | -0.04 (-0.08, -0.01) | -0.03 (-0.07, 0.00) |
| GW 0-12 | 2974 | -0.01 (-0.05, 0.02) | 0.02 (-0.02, 0.06) |
| GW >12 | 2138 | -0.05 (-0.09, -0.01) | -0.03 (-0.07, 0.02) |
|  |  |  |  |
| **Mapping test - Reading** | 38,659 | Odds ratio (95% CI) | Odds ratio (95% CI) |
|  |  |  |  |
| Any iodine supplement use GW 0-22 | 14,350 | 0.93 (0.89, 0.98) | 0.98 (0.94, 1.03) |
| First report of iodine supplement |  |  |  |
| Never (non-supplement user) | 24,309 | 1 (ref.) | 1 (ref.) |
| Before pregnancy^c^ | 4,021 | 0.96 (0.89, 1.03) | 1.02 (0.95, 1.11) |
| GW 0-12 | 3461 | 0.97 (0.90, 1.05) | 1.02 (0.94, 1.11) |
| GW >12 | 2589 | 0.88 (0.81, 0.97) | 0.91 (0.83, 1.00) |
|  |  |  |  |
| **Mapping test – Mathematics** | 37,433 | Odds ratio (95% CI) | Odds ratio (95% CI) |
|  |  |  |  |
| Any iodine supplement use GW 0-22 | 13,906 | 0.97 (0.92, 1.03) | 0.99 (0.93, 1.04) |
| First report of iodine supplement |  |  |  |
| Never (non-supplement user) | 23,527 | 1 (ref.) | 1 (ref.) |
| Before pregnancy^c^ | 3887 | 0.92 (0.84, 1.01) | 0.96 (0.88, 1.06) |
| GW 0-12 | 3366 | 1.02 (0.93, 1.12) | 1.04 (0.94, 1.14) |
| GW >12 | 2509 | 0.95 (0.85, 1.06) | 0.97 (0.87, 1.08) |
|  |  |  |  |
| **Special education** | 39,471 | Odds ratio (95% CI) | Odds ratio (95% CI) |
|  |  |  |  |
| Any iodine supplement use GW 0-22 | 14,665 | 0.92 (0.85, 1.00) | 0.96 (0.88, 1.04) |
| First report of iodine supplement |  |  |  |
| Never (non-supplement user) | 24,806 | 1 (ref.) | 1 (ref.) |
| Before pregnancy^c^ | 4094 | 0.89 (0.78, 1.02) | 0.95 (0.83, 1.09) |
| GW 0-12 | 3540 | 0.98 (0.85, 1.12) | 1.03 (0.89, 1.18) |
| GW >12 | 2653 | 0.85 (0.72, 1.01) | 0.87 (0.73, 1.02) |

Standardized beta>0 and odds ratio>1 indicate poorer performance or increased likelihood of special education.
^a^ All models (including crude models) were adjusted for random effects of sibling clusters. ^b^ Models were adjusted for maternal age, BMI, parity, education, smoking in pregnancy, fibre intake, EPA and DHA intake, folic acid supplement within the interval from 4 weeks before to 8 weeks after conception, child sex (only models with continuous outcomes), bilingual parent(s) (only language outcome), and maternal history of reading/writing difficulty (only continuous outcomes for reading and writing skills).
^c^ 1-26 weeks before conception
